# Limited Efficacy of Anti-EGFR Monoclonal Antibodies in Colorectal Cancer Patients with Rare RAS Variants: Analysis of the C-CAT Database

**DOI:** 10.3390/cimb46120869

**Published:** 2024-12-23

**Authors:** Shuhei Suzuki, Yosuke Saito, Koki Saito, Yuta Yamada, Koshi Takahashi, Ryosuke Kumanishi, Tadahisa Fukui, Takashi Yoshioka

**Affiliations:** 1 Yamagata Hereditary Tumor Research Center, Yamagata University, 1-4-12 Kojirakawa, Yamagata 990-8560, Japan; 2 Department of Clinical Oncology, School of Medicine, Yamagata University, 2-2-2 Iida-Nishi, Yamagata 990-9585, Japan; 3 Department of Gastroenterology, Yamagata City Hospital Saiseikan, 1-2-26 Nanokamachi, Yamagata 990-0042, Japan; 4 Graduate School of Medicine, Tokyo Medical University, 6-1-1 Shinjuku, Tokyo 160-8402, Japan

**Keywords:** colorectal cancer, rare variants, *KRAS*, *NRAS*, genomic testing, cetuximab, panitumumab

## Abstract

Epidermal growth factor receptor (EGFR) inhibition is crucial in treating RAS wild-type metastatic colorectal cancer, yet current testing methods may miss rare RAS variants affecting treatment efficacy. We analyzed 4122 colorectal cancer patients receiving anti-EGFR antibodies from the Center for Cancer Genomics and Advanced Therapeutics database, identifying 54 patients (1.3%) with rare RAS variants undetectable by standard testing. These patients showed significantly lower response rates to anti-EGFR therapy (28.3%) compared to RAS wild-type cases (44.6%, *p* = 0.003). Disease control rates were also lower in rare variant cases (60.9%) versus wild-type cases (80.0%). Most common rare variants included *KRAS* Q22K, A59E, and A11_G12insGA. Comprehensive genomic profiling revealed additional alterations in *TP53* (90.7%), *APC* (87.0%), and non-V600E *BRAF* mutations (25.9%). Our findings suggest that rare RAS variants predict poor anti-EGFR therapy response, highlighting the potential benefit of comprehensive genomic profiling before treatment initiation. This study provides real-world evidence supporting the clinical relevance of rare RAS variants in treatment decision-making for colorectal cancer. Future studies should focus on developing cost-effective comprehensive testing strategies and evaluating alternative treatment approaches for patients with rare RAS variants.

## 1. Introduction

Epidermal growth factor receptor (EGFR) inhibition with monoclonal antibodies represents a cornerstone of treatment for RAS wild-type metastatic colorectal cancer [1,2,3,4]. The emergence of anti-EGFR therapies has significantly improved survival outcomes in metastatic colorectal cancer patients, with high response rates in RAS wild-type cases [1,3]. The efficacy of anti-EGFR therapy is strongly dependent on the absence of RAS pathway alterations, making RAS testing mandatory before treatment initiation [1,4].

Current standard testing methods in many healthcare systems, including Japan, are limited to detecting common mutations. Common mutations include *KRAS* G12D, G12V, and G13D mutations in exon 2, which together account for approximately two-thirds of all RAS mutations in colorectal cancer. These mutations primarily occur in *KRAS* (30–35%) and *NRAS* (3–5%), with specific hotspots in codons 12, 13, and 61 being most common. This limitation stems from both technological constraints and healthcare policy decisions, potentially leading to suboptimal treatment selection. The molecular landscape of colorectal cancer is complex, with RAS mutations representing the most frequent alterations, occurring in approximately 40% of cases [5,6]. Understanding the full spectrum of these mutations, including rare variants, has become increasingly important for optimal treatment selection.

The MEBGEN RASKET-B kit, commonly used in Japan, detects specific mutations in *KRAS*/*NRAS* exons 2, 3, and 4, while the OncoBEAM RAS CRC kit covers a similar spectrum of mutations. These methods have proven reliable for detecting common RAS variants but may miss rare alterations that could affect treatment efficacy.

Comprehensive genomic profiling (CGP) has emerged as a powerful tool capable of identifying these rare variants. However, insurance coverage for CGP is typically limited to later-line settings in some circumstances [7]. This restriction creates a potential gap in optimal treatment selection, particularly in the first-line setting, where treatment decisions have the most significant impact on patient outcomes [8]. The ability to detect rare RAS variants through CGP could prevent the administration of potentially ineffective treatments and guide more appropriate therapeutic choices.

Recent studies have suggested that rare RAS variants may have similar biological effects to common mutations in terms of constitutive pathway activation and resistance to anti-EGFR therapy [9]. However, the real-world prevalence and clinical impact of these rare variants remain poorly characterized, particularly in Asian populations.

## 2. Materials and Methods

### 2.1. Database and Patient Selection

The C-CAT database (ver. 20240419) represents Japan’s largest cancer genomics repository [10], containing comprehensive molecular profiling data from 71,779 cases across multiple cancer types. Within this database, colorectal cancer cases number 11,992, providing a robust dataset for analysis. The database incorporates results from five distinct genomic profiling platforms: the NCC Oncopanel System, FoundationOne CDx, FoundationOne Liquid CDx, Guardant360 CDx, and GeneMine^TM^ TOP Cancer Panel, each employed during specific time periods from June 2019 to April 2024.

These platforms offer varying capabilities in terms of gene coverage, specimen requirements, and additional molecular features analysis. The NCC Oncopanel System examines 114 genes and includes microsatellite instability testing in its newer versions, while FoundationOne CDx provides comprehensive coverage of 324 genes with integrated Microsatellite Instability (MSI) and Tumor Mutational Burden (TMB) assessment. FoundationOne Liquid CDx offers blood-based testing with identical gene coverage to its tissue-based counterpart, particularly valuable for patients unable to undergo tissue biopsy. Guardant360 CDx, though more focused with 73 genes, specializes in rapid blood-based analysis, while the GeneMine^TM^ TOP Cancer Panel offers the most extensive coverage with 723 genes.

### 2.2. Definition of Pathogenic RAS Variants

Our approach to RAS variant classification followed a comprehensive methodology integrating multiple layers of evidence. Variants were initially identified as those not detectable by current standard testing methods (RASKET-B or OncoBEAM) and subsequently evaluated for pathogenicity through the OncoKB database (https://www.oncokb.org/, accessed on 4 May 2024). Following the instructions of the genetics expert, we classified variants as pathogenic when designated as Likely Oncogenic or higher in OncoKB, and additionally included *KRAS* T20M based on previously published functional studies [9]. In cases where multiple alterations were detected, we prioritized frameshift mutations over point mutations, and mutations over amplifications. To maintain analytical clarity, cases harboring concurrent common RAS mutations were excluded from the analysis.

### 2.3. Clinical Data Collection and Outcome Assessment

As previously described [11], we conducted a retrospective cohort study collecting comprehensive clinical, pathological, and genomic data. In brief, we gathered demographic information, pathological characteristics including histological type and tumor content, and detailed clinical sample information. Treatment histories, responses, and lifestyle factors were documented alongside family history of malignancy. Genomic testing results underwent standardized review by Expert Panels comprising medical oncologists, pathologists, clinical geneticists, bioinformaticians, and treating physicians, in accordance with Japanese insurance requirements.

Genomic analysis included evaluation of gene calls, tumor mutational burden, microsatellite instability status, and specific genetic alterations. Variant pathogenicity was assessed using established databases (ClinVar, OncoKB, jMorp), with variants classified as ’Likely Oncogenic’ or higher in OncoKB, or meeting C-CAT evidence level F or higher being considered pathogenic. Treatment responses were documented by physicians at each institution with reference to Response Evaluation Criteria in Solid Tumors (RECIST), with outcomes categorized as complete response, partial response, stable disease, progressive disease, or not evaluated.

### 2.4. Statistical Analysis

Exploratory statistical analyses were performed using Microsoft Excel 2021 and Statcel 5 (OMS Publishing Inc., Saitama, Japan). This exploratory analysis focused on identifying potential associations between genetic alterations and treatment responses. Categorical variables were analyzed using chi-square tests. Response rate was defined as complete response and partial response, while disease control rate included complete response, partial response, and stable disease. All statistical tests were two-sided, with findings considered exploratory in nature given the limited sample size of this rare tumor cohort. Missing data were excluded from the analysis without imputation, considering the retrospective nature of the study. Statistical analyses included calculation of 95% confidence intervals for all response rates. Given the relatively small sample size of rare variant cases, results were interpreted with appropriate caution, and statistical limitations were explicitly acknowledged.

## 3. Results

### 3.1. Patient Population Characteristics

Among the 71,779 cases registered in the C-CAT database (Table 1), colorectal cancer represented 11,992 cases (16.7%). In this cohort, 4122 patients received anti-EGFR antibody treatment, with 54 patients (1.3%) identified as harboring rare RAS variants (Table 2). These rare variants were detected using MEBGEN RASKET™-B Kit (MBL, Nagoya, Japan) and OncoBEAM™ RAS CRC Kit (Sysmex, Kobe, Japan), with the specific genes tested listed in Table 3, as described in the Section 2. The median age of patients with rare RAS variants was 62 years (range: 32–79 years), with the majority falling into the 60–69 years age group (*n* = 20), followed by 50–59 years (*n* = 16), and 70–79 years (*n* = 14). The gender distribution was equal (27 males and 27 females).

Genomic profiling was predominantly conducted using FoundationOne CDx (Foundation Medicine, Cambridge, MA, USA) (*n* = 44), followed by NCC Oncopanel System (Sysmex) (*n* = 6), FoundationOne Liquid CDx (Foundation Medicine) (*n* = 3), and Guardant360 CDx (Guardant Health, Redwood City, CA, USA) (*n* = 1). Testing specimens were obtained from primary sites (*n* = 37), metastatic sites (*n* = 13), and blood samples (*n* = 4) (Table 4).

Regarding Eastern Cooperative Oncology Group Performance Status (ECOG PS), most patients demonstrated favorable performance status, with 30 patients (55.6%) classified as PS 0, 22 patients (40.7%) as PS 1, and 2 patients (3.7%) as PS 2. The majority of patients (*n* = 49, 90.7%) received anti-EGFR therapy in combination with cytotoxic agents. Among these patients, panitumumab (*n* = 40) was more commonly used than cetuximab (*n* = 14).

The distribution of metastatic sites showed a pattern typical of advanced colorectal cancer, with liver metastases being most frequent (*n* = 36, 66.7%), followed by lung metastases (*n* = 30, 55.6%), peritoneal dissemination (*n* = 15, 27.8%), bone metastases (*n* = 5, 9.3%), and other sites (*n* = 20, 37.0%). Regarding lifestyle factors, 24 patients had a smoking history and 8 patients reported a history of excessive alcohol consumption. Family history of cancer was present in 38 patients.

### 3.2. Overview of Genomic Testing Results

We analyzed genetic alterations identified through cancer genome profiling tests in 54 colorectal cancer cases. The TMB ranged from 0 to 246 mutations/Mb, with a mean TMB of 4 mutations/Mb.

**Figure 1:**
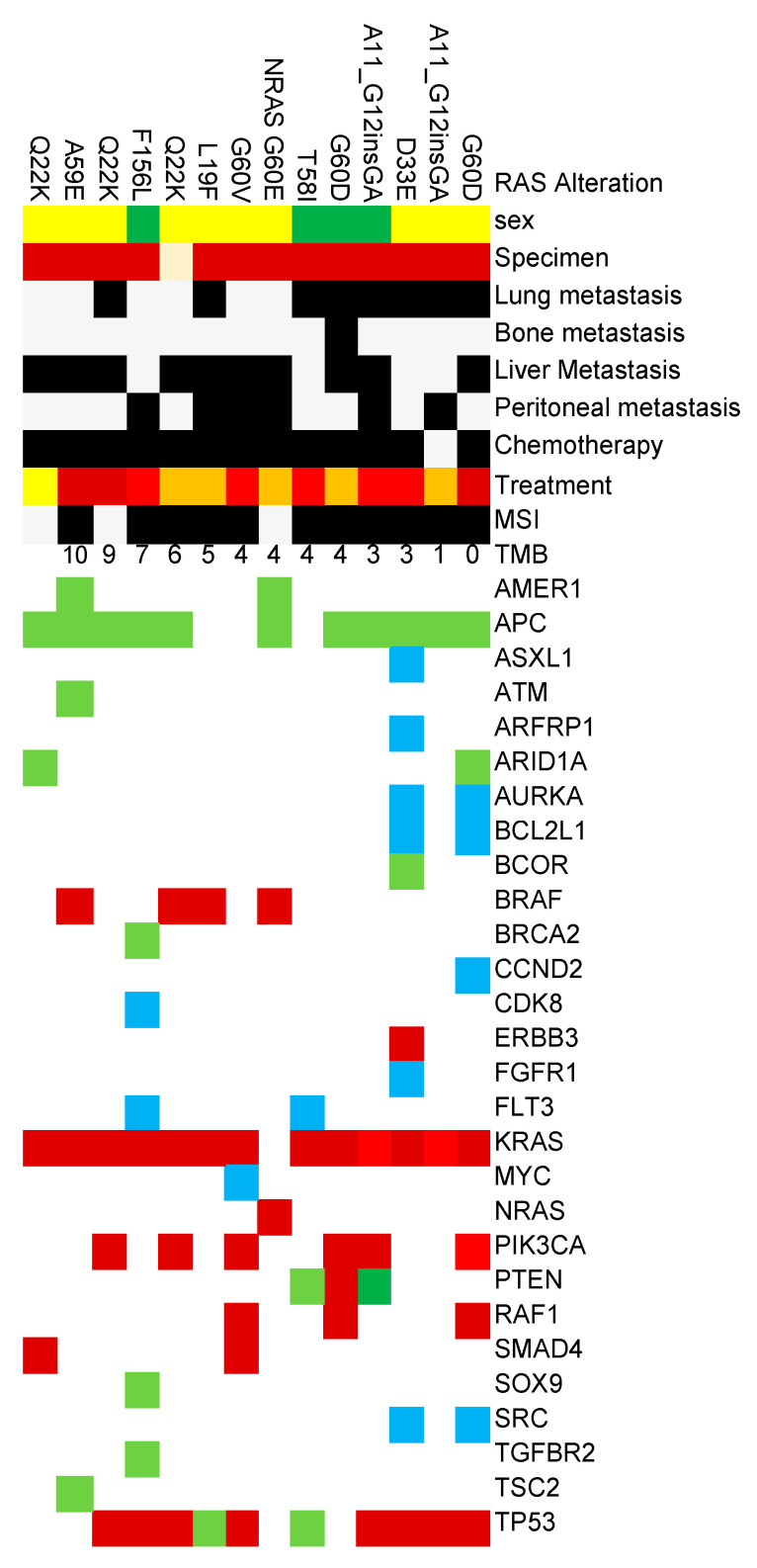

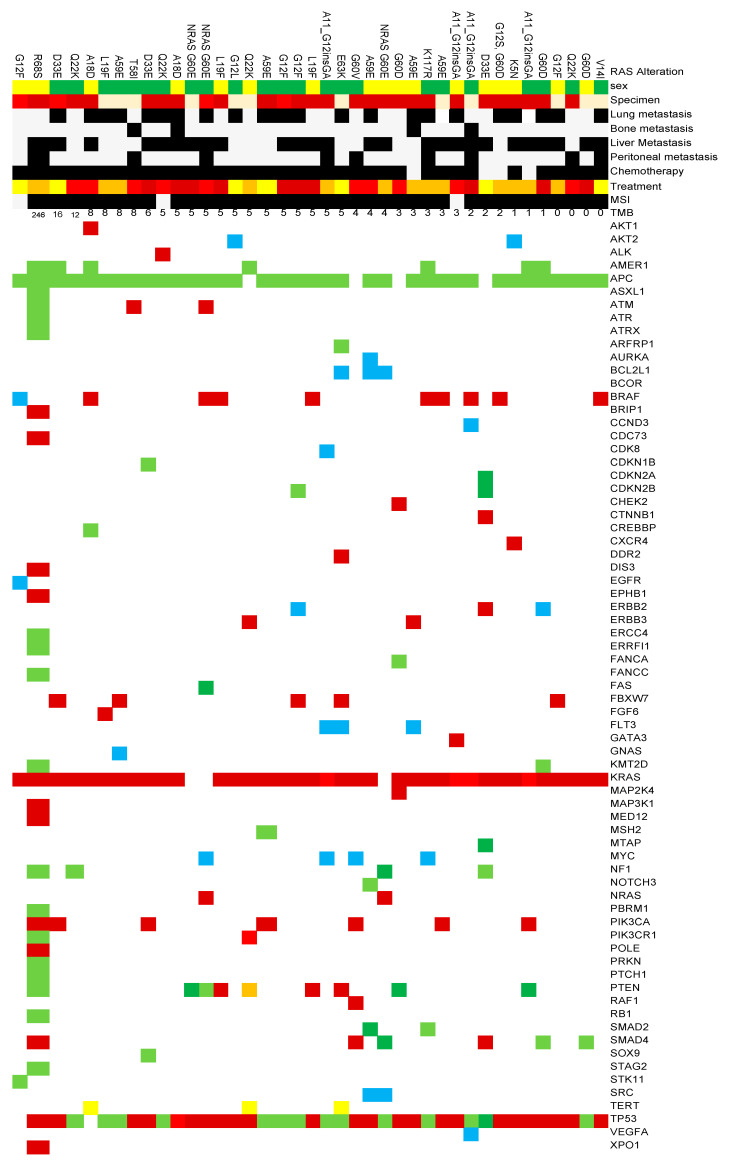

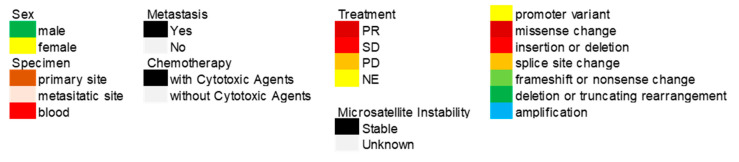
Genomic alterations and anti-EGFR antibodies (Upper, cetuximab; Lower, Panitumumab) treatment responses of rare RAS variants cases registered in the Center for Cancer Genomics and Advanced Therapeutics. TMB: Tumor Mutational Burden; PR: Partial Response; SD: Stable Disease; PD: Progressive Disease; NE: not evaluated.

The most frequently observed alterations were in *TP53* and *APC* genes, detected in 49 (90.7%) and 47 (87.0%) cases, respectively, indicating their fundamental role in colorectal carcinogenesis. These were followed by non-V600E *BRAF* mutations in 14 cases (25.9%), *PIK3CA* alterations in 13 cases (24.1%), and *PTEN* alterations in 12 cases (22.2%) (Figure 1).

Several other oncogenic alterations were detected at lower frequencies, including alterations in *ERBB2* and *MYC* genes. These alterations may have implications for targeted therapy selection and prognosis.

Regarding microsatellite stability status, 48 cases (88.9%) were microsatellite stable (MSS), while 6 cases (11.1%) had unknown status.

Among rare RAS variants undetectable by routine clinical testing kits, *KRAS* Q22K was relatively frequently registered with 7 cases, followed by *KRAS* A59E and *KRAS* A11_G12insGA with 6 cases each, *KRAS* G60D with 5 cases, and *KRAS* G33E with 4 cases.

### 3.3. Treatment Outcomes Analysis

The analysis of treatment outcomes revealed striking differences between patients with rare RAS variants and other subgroups. The objective response rate in patients with rare RAS variants showed a tendency to be lower at 28.3% (95% Confidence Interval: 15.2–41.3%) compared to 44.6% in RAS wild-type patients and 38.1% (95% Confidence Interval: 43.0–46.2%) in patients with common RAS mutations, with varying levels of statistical evidence (*p* = 0.003 and *p* = 0.090, respectively, Table 5). This pattern was further reflected in the disease control rates, which showed marked disparities between rare RAS variant cases (60.9%), RAS wild-type cases (80.0%), and common mutation cases (74.8%).

## 4. Discussion

Our analysis of the C-CAT database provides several crucial insights into the clinical significance of rare RAS variants in colorectal cancer treatment. The identification of rare RAS variants in 1.3% of patients receiving anti-EGFR therapy represents a significant finding, as these patients’ received treatments that might have been avoided with comprehensive pre-treatment molecular testing. The substantially lower response rates observed in these patients (28.3% versus 44.6% in RAS wild-type cases) underscores the clinical relevance of identifying these variants before treatment initiation.

The molecular heterogeneity of colorectal cancer has been increasingly recognized, but the impact of rare genomic alterations on treatment outcomes has remained poorly characterized. Our findings build upon previous work by Loree et al. [9], who identified the functional significance of rare RAS variants in preclinical models. The present study provides real-world validation of these laboratory findings, demonstrating that rare RAS variants indeed predict poor response to anti-EGFR therapy in clinical practice.

The disparity in response rates between patients with rare variants and those with RAS wild-type status raises important questions about current testing strategies. While the RASKET-B and OncoBEAM platforms have served as valuable tools for identifying common RAS mutations, our data suggest that their limited scope may result in missed opportunities for optimal treatment selection. The economic implications of this finding are substantial, considering the high cost of anti-EGFR antibodies [12] and the potential for unnecessary toxicity exposure in patients unlikely to benefit from treatment.

The timing of CGP testing emerges as a critical consideration from our analysis. Current healthcare policies in many circumstances, including Japan, restrict CGP coverage to later-line settings [7]. This approach may be counterproductive, as our data suggest that earlier comprehensive testing might help optimize treatment selection and guide more appropriate therapeutic choices from the outset. The cost-effectiveness of implementing CGP before first-line therapy warrants careful evaluation, considering both the direct costs of testing and the potential savings from avoiding less optimal treatments.

Our findings also have implications for clinical trial design and biomarker development. The significant impact of rare RAS variants on treatment outcomes suggests that future trials of EGFR-targeted therapies should consider comprehensive molecular profiling in their eligibility criteria. Additionally, the development of more comprehensive companion diagnostic tests that can detect these rare variants may be necessary to optimize patient selection for anti-EGFR therapy.

Several limitations of our study warrant discussion. First, our analysis was based on amino acid changes without confirmation of specific nucleotide alterations, which could potentially mask some molecular complexity. Second, our inclusion of gastrointestinal tract cancers broadly, incorporating appendiceal and small intestinal cancers, may introduce some heterogeneity into the analysis. However, this approach reflects real-world practice patterns where these cancers are often treated similarly to colorectal cancer [13]. Third, the timing of specimen collection was not considered in our analysis, and some liquid biopsy results may reflect acquired resistance mutations rather than primary variants. Finally, we did not analyze the impact of chemotherapy backbone or treatment line on outcomes, which could potentially influence response rates.

Despite these limitations, our findings suggest evidence for the clinical relevance of rare RAS variants in colorectal cancer. The lower response rates observed in patients with these variants indicate that current standard testing methods may benefit from additional consideration for optimal patient selection for anti-EGFR therapy. The implementation of CGP before first-line therapy might contribute to treatment outcomes through more comprehensive patient selection and appropriate treatment allocation.

The implications of our findings extend beyond individual patient care to healthcare policy and resource allocation. The current paradigm of reserving CGP for later-line treatment may need reconsideration in light of these results. While the upfront costs of implementing CGP in the first-line setting would be substantial, the potential benefits of optimized treatment selection could offset these costs through improved outcomes and avoided ineffective therapies. A comprehensive cost-effectiveness analysis would need to consider not only the direct costs of testing and treatment but also the indirect costs associated with managing adverse events and disease progression in patients receiving suboptimal therapy.

The evolution of molecular testing in colorectal cancer reflects a broader trend toward precision oncology, but our findings highlight persistent gaps in current approaches. The identification of rare RAS variants through CGP represents an opportunity to further refine patient selection for anti-EGFR therapy, potentially improving the therapeutic index of these agents. This refinement becomes particularly important as the treatment landscape for colorectal cancer continues to evolve, with new targeted therapies and immunotherapy approaches emerging [14,15].

From a biological perspective, rare RAS variants can affect RAS protein function through various mechanisms, including altered GTP binding, impaired GTPase activity, or modified interactions with downstream effectors. These alterations can lead to constitutive pathway activation similar to common mutations, potentially conferring resistance to anti-EGFR therapy. Our results support the hypothesis that rare RAS variants can have functional consequences similar to common mutations in terms of pathway activation and treatment resistance. This finding aligns with structural biology studies demonstrating that various RAS alterations can lead to similar conformational changes and downstream signaling effects [9]. The lower response rates observed in our study might provide clinical validation of these mechanistic insights.

Future research directions suggested by our findings include the need for prospective validation of comprehensive molecular testing strategies, investigation of potential differences in the biology of rare versus common RAS variants, and evaluation of alternative treatment approaches for patients harboring these variants. Additionally, the development of more comprehensive, cost-effective testing platforms that can detect both common and rare variants could help bridge the current gap between standard testing and CGP.

## 5. Conclusions

This analysis of the C-CAT database demonstrates that rare RAS variants, detectable only through comprehensive genomic profiling, are associated with significantly poorer outcomes in patients receiving anti-EGFR therapy for colorectal cancer. The current practice of limiting CGP to later-line settings may result in suboptimal treatment selection for patients harboring these variants. Our findings support the implementation of more comprehensive molecular testing before first-line therapy to improve patient selection and treatment outcomes. While several limitations must be acknowledged, the clinical implications of our results warrant serious consideration of changes to current testing and treatment algorithms. Further research is needed to validate these findings prospectively and to evaluate the cost-effectiveness of earlier CGP implementation in the treatment paradigm for colorectal cancer.

## Figures and Tables

**Table 1:** Background of registered cases in the Center for Cancer Genomics and Advanced Therapeutics in Japan.

Total Cases (*n* = 71,779)			
Primary Site		Cancer Genomics Test	
Colorectal	11,992	FoundationOne CDx	51,944
Pancreas	10,694	FoundationOne Liquid CDx	10,751
Bile Duct	6207	NCC Oncopanel System	7443
Esophagus/Stomach	4448	GenMine^TM^ TOP Cancer Panel	915
Prostate	4423	Guardant360 CDx	726
Breast	4270		
Lung	4235	Representative Gene Alterations	
Ovary/Fallopian Yube	4098	*TP53*	42,998
Soft Tissue	2962	*KRAS*	19,880
Uterus	2566	*CDKN2A*	14,807
Others	15,884	*APC*	14,717
Sex			
Male	36,348		
Female	35,426		
Unknown	5		

The study period for each cancer genomic testing was as follows: NCC Oncopanel System (1 June 2019 to 17 April 2024), FoundationOne^®^ CDx (1 June 2019 to 17 April 2024), FoundationOne^®^ Liquid CDx (1 August 2021 to 17 April 2024), Guardant360^®^ CDx (24 July 2023 to 17 April 2024), and GeneMineTM TOP Cancer Panel (1 August 2023 to 17 April 2024).

**Table 2:** Background of rare RAS Alteration Cases treated with anti-EGFR antibodies registered in the Center for Cancer Genomics and Advanced Therapeutics.

Total Rare RAS Variant Cases Treated with Anti-EGFR Antibodies (*n* = 54)			
Age (years; median 62, range 32–79)		Cancer Testing Panel	
60–69	20	FoundationOne CDx	44
50–59	16	NCC Oncopanel System	6
70–79	14	FoundationOne Liquid CDx	3
40–49	3	Guardant 360 CDx	1
30–39	1		
		ECOG PS	
Sex		0	30
Male	27	1	22
Female	27	2	2
Smoking History		Metastatic Sites	
No	28	Liver	36
Yes	24	Lung	30
Unknown	2	Peritoneal	15
		Bone	5
Drinking History		Other Sites	20
No	41		
Yes	8	Anti-EGFR Antibodies (Earlier Line)	
Unknown	5	Panitumumab	40
		Cetuximab	14
Family History (Cancer)			
Yes	38	Concomitant Use of Cytotoxic Agents	
No	16	Yes	49
		No	5
Specimen			
Primary Site	37		
Metastatic Site	13		
Blood	4		

ECOG PS: Eastern Cooperative Oncology Group Performance Status.

**Table 3:** Detectable gene mutations using MEBGEN RASKET™-B Kit and OncoBEAM™ RAS CRC Kit.

MEBGEN RASKET™-B Kit		
*KRAS*	Exon2	G12S, G12C, G12R, G12D, G12V, G12A, G13S, G13C, G13R, G13D, G13V, G13A
	Exon3	A59T, A59G, Q61K, Q61E, Q61L, Q61P, Q61R, Q61H
	Exon4	K117N, A146T, A146P, A146V
*NRAS*	Exon2	G12S, G12C, G12R, G12D, G12V, G12A, G13S, G13R, G13D, G13V, G13A
	Exon3	A59T, A59G, Q61K, Q61E, Q61L, Q61P, Q61R, Q61H
	Exon4	K117N, A146T, A146P, A146V
*BRAF*	Exon15	V600E
OncoBEAM™ RAS CRC Kit		
*KRAS*	Exon2	G12S, G12C, G12R, G12D, G12V, G12A, G13D
	Exon3	A59T, Q61L, Q61R, Q61H
	Exon4	K117N, A146T, A146V
*NRAS*	Exon2	G12S, G12C, G12R, G12D, G12V, G12A, G13R, G13D, G13V
	Exon3	A59T, Q61K, Q61L, Q61R, Q61H
	Exon4	K117N, A146T

**Table 4:** List of genomic tests covered by insurance in Japan.

Features	FoundationOne CDx	FoundationOne Liquid CDx	NCC Oncopanel System	Guardant 360 CDx	GenMine TOP Cancer Panel
Sample Type	FFPE tissue	FFPE tissue	FFPE tissue	Blood	FFPE tissue
Number of Genes	324	324	114	74	723
MSI Testing	Yes	Yes	Yes *	Yes	No
TMB Assesment	Yes	Yes	Yes	No	Yes
Minimum Tumor Content Required	20%	N/A	20%	N/A	20%
Required DNA Input	50 ng	2 tubes	50 ng	2 tubes **	50 ng

* Earlier models did not include MSI testing capability. ** Including a spare tube. FFPE: Formalin-Fixed Paraffin-Embedded, MSI: Microsatellite Instability, TMB: Tumor Mutational Burden, N/A: Not Applicable.

**Table 5:** Treatment Response to anti-EGFR Treatments.

Treatment Response to anti-EGFR Treatments		Response Rate to anti-EGFR Treatments	
Partial Response	13	All Cases (*n* = 4122)	43.6%
Stable Disease	15	RAS Wildtype Cases (*n* = 3513)	44.6%
Progressive Disease	18	RAS Mutation Cases (*n* = 611)	38.1%
Not Evaluated	8	Rare RAS Alteration Cases (*n* = 54)	28.3%
Microsatellite Instability		Disease Control Rate to anti-EGFR Treatments	
Microsatellite Stable	48	All Cases (*n* = 4122)	79.2%
Unknown	6	RAS Wildtype Cases (*n* = 3513)	80.0%
		RAS Mutation Cases (*n* = 611)	74.8%
		Rare RAS Alteration Cases (*n* = 54)	60.9%

## Data Availability

Data are contained within the article.

## References

[1] Amado R.G., Wolf M., Peeters M., Van Cutsem E., Siena S., Freeman D.J., Juan T., Sikorski R., Suggs S., Radinsky R. (2008). Wild-type KRAS is required for panitumumab efficacy in patients with metastatic colorectal cancer. J. Clin. Oncol..

[2] Schwartzberg L.S., Rivera F., Karthaus M., Fasola G., Canon J.L., Hecht J.R., Yu H., Oliner K.S., Go W.Y. (2014). PEAK: A randomized, multicenter phase II study of panitumumab plus modified fluorouracil, leucovorin, and oxaliplatin (mFOLFOX6) or bevacizumab plus mFOLFOX6 in patients with previously untreated, unresectable, wild-type KRAS exon 2 metastatic colorectal cancer. J. Clin. Oncol..

[3] Watanabe J., Muro K., Shitara K., Yamazaki K., Shiozawa M., Ohori H., Takashima A., Yokota M., Makiyama A., Akazawa N. (2023). Panitumumab vs Bevacizumab Added to Standard First-line Chemotherapy and Overall Survival Among Patients With RAS Wild-type, Left-Sided Metastatic Colorectal Cancer: A Randomized Clinical Trial. JAMA.

[4] Chen D., Li L., Zhang X., Gao G., Shen L., Hu J., Yang M., Liu B., Qian X. (2018). FOLFOX plus anti-epidermal growth factor receptor (EGFR) monoclonal antibody (mAb) is an effective first-line treatment for patients with RAS-wild left-sided metastatic colorectal cancer: A meta-analysis. Medicine.

[5] Osumi H., Shinozaki E., Suenaga M., Matsusaka S., Konishi T., Akiyoshi T., Fujimoto Y., Nagayama S., Fukunaga Y., Ueno M. (2016). RAS mutation is a prognostic biomarker in colorectal cancer patients with metastasectomy. Int. J. Cancer.

[6] Bylsma L.C., Gillezeau C., Garawin T.A., Kelsh M.A., Fryzek J.P., Sangaré L., Lowe K.A. (2020). Prevalence of RAS and BRAF mutations in metastatic colorectal cancer patients by tumor sidedness: A systematic review and meta-analysis. Cancer Med..

[7] Ebi H., Bando H. (2019). Precision Oncology and the Universal Health Coverage System in Japan. JCO Precis. Oncol..

[8] Matsubara J., Mukai K., Kondo T., Yoshioka M., Kage H., Oda K., Kudo R., Ikeda S., Ebi H., Muro K. (2023). First-Line Genomic Profiling in Previously Untreated Advanced Solid Tumors for Identification of Targeted Therapy Opportunities. JAMA Netw. Open.

[9] Loree J.M., Wang Y., Syed M.A., Sorokin A.V., Coker O., Xiu J., Weinberg B.A., Vanderwalde A.M., Tesfaye A., Raymond V.M. (2021). Clinical and Functional Characterization of Atypical KRAS/NRAS Mutations in Metastatic Colorectal Cancer. Clin. Cancer Res..

[10] Kohno T., Kato M., Kohsaka S., Sudo T., Tamai I., Shiraishi Y., Okuma Y., Ogasawara D., Suzuki T., Yoshida T. (2022). C-CAT: The National Datacenter for Cancer Genomic Medicine in Japan. Cancer Discov..

[11] Suzuki S., Saito Y. (2024). Genomic Analysis of Advanced Phyllodes Tumors Using Next-Generation Sequencing and Their Chemotherapy Response: A Retrospective Study Using the C-CAT Database. Medicina.

[12] Carvalho A.C., Leal F., Sasse A.D. (2017). Cost-effectiveness of cetuximab and panitumumab for chemotherapy-refractory metastatic colorectal cancer. PLoS ONE.

[13] de Back T., Nijskens I., Schafrat P., Chalabi M., Kazemier G., Vermeulen L., Sommeijer D. (2023). Evaluation of Systemic Treatments of Small Intestinal Adenocarcinomas: A Systematic Review and Meta-analysis. JAMA Netw. Open.

[14] Johnson D., Chee C.E., Wong W., Lam R.C.T., Tan I.B.H., Ma B.B.Y. (2024). Current advances in targeted therapy for metastatic colorectal cancer-Clinical translation and future directions. Cancer Treat. Rev..

[15] Bando H., Ohtsu A., Yoshino T. (2023). Therapeutic landscape and future direction of metastatic colorectal cancer. Nat. Rev. Gastroenterol. Hepatol..

